# Resolution effects in reconstructing ancestral genomes

**DOI:** 10.1186/s12864-018-4462-y

**Published:** 2018-05-09

**Authors:** Chunfang Zheng, Yuji Jeong, Madisyn Gabrielle Turcotte, David Sankoff

**Affiliations:** Department of Mathematics and Statistics, University of Ottawa, 585 King Edward Avenue, Ottawa, Ontario, K1N 6N5 Canada

**Keywords:** Granularity, Gentianales, Gene order, Median, Synteny blocks, Rearrangements

## Abstract

**Background:**

The reconstruction of ancestral genomes must deal with the problem of resolution, necessarily involving a trade-off between trying to identify genomic details and being overwhelmed by noise at higher resolutions.

**Results:**

We use the median reconstruction at the synteny block level, of the ancestral genome of the order Gentianales, based on coffee, *Rhazya stricta* and grape, to exemplify the effects of resolution (granularity) on comparative genomic analyses.

**Conclusions:**

We show how decreased resolution blurs the differences between evolving genomes, with respect to rate, mutational process and other characteristics.

## Background

All comparative genomics, including the reconstruction of ancestral genomes must deal, implicitly or explicitly, with the problem of resolution, sometimes called “granularity”, a topic that has preoccupied us since the mid-2000s (e.g., [1]), after the publication of the human and mouse genomes, and increasingly with the next few mammalian genomes [2].

Setting the level of resolution involves a trade-off between trying to decipher precise genomic details on one hand and being overwhelmed by noise at higher resolutions due to both mutational processes and methodological difficulties, on the other. Thus, despite some successes, notably with the ancient “boreoeutherian” mammalian genome [3], in most cases, a nucleotide by nucleotide reconstruction of even the coding region of an ancestral gene contains a large amount of uncertainty, while non-coding DNA reconstruction is much more refractory.

Starting few years ago, a number of algorithmic approaches to these questions have been proposed (e.g., [4–10], as reviewed by [11]).

At the level of gene order, especially in plants, the processes of whole genome duplication and fractionation compound inherent inferential difficulties due to genome rearrangement. A telling aspect in the widely-used synteny block identifier SYNMAP [12, 13] is the default requirement that at least five pairs of orthologous genes in two genomes or five pairs of paralogous genes in a single genome, in close succession, are necessary before we have confidence that the two apparently homologous segments containing these genes are genuinely contemporaneous descendants of the same ancestral genomic fragment.

In this paper, we explore the difficulty in establishing synteny blocks for three genomes based on the pairwise output of SYNMAP, as a preliminary to a stepwise decrease in resolution in the calculation of the median estimate of the ancestor. (The median is a reconstructed genome that minimizes the sum of some distance measure from itself to three or more given genomes.)

There are a number of ways (e.g. [2, 4–11]) of establishing synteny blocks for more than two genomes and of changing the resolution of the analysis. Since our main goal in this paper is to detail the *consequences* of the loss of resolution, we do not make the assumptions or adopt the objective functions of any of these. Rather we carefully monitor the merger or split of overlapping pairwise synteny blocks, which may coincide on each genome without necessarily containing any genes in common. This procedure serves as preprocessing for the main exercise, which starts with the construction, at eight levels of resolution, of the median of three genomes, two in the order Gentianales: coffee (*Coffea canephora*, family Rubiaceae) [14] and *Rhazya stricta* (family Apocynaceae) [15], and one rosid outgroup: grape (*Vitis vinifera*, family Vitaceae, order Vitales) [16]. The interest in the two Gentianales species stems from our participation in the recent genome sequencing projects of both, and the fact that they are among the only sequenced asterid genomes to have escaped any whole genome duplication or triplication events more recent than the core eudicot triplication known as *γ*. This is true also of the conservative rosid, grape, which allows us to avoid reconstruction difficulties due to recently generated paralogous synteny blocks. The assemblies of the grape and coffee genomes are available in CoGe [12, 13] at the pseudo-molecule level, while the *Rhazya* scaffolds are of equal or better quality, but lack any ancillary data that could enable pseudo-molecule construction.

Each median construction is comprehensively analyzed in comparison with those of greater and lesser resolution, using DCJ (double-cut-and-join) distance [17], breakpoint distance, and a measure of interchromosomal mixing [1]. This analysis reveals the effects of resolution on the estimates of total median distance (i.e., the sum of the distances between the median and the three input genomes), the differential between the rates of gene-order evolution of the three plant genomes, the relative preponderance of translocation, reversals and other rearrangement mutations, and the breakpoint reuse rate. In addition, at full resolution, this work provides a detailed reconstruction of the ancestral Gentianales genome at the synteny block level.

## Methods

The whole genome triplication *γ*, discovered by Jaillon et al. [16] while sequencing the grape genome, is broadly accepted to have occurred at the root of the core eudicots. This polyploidization produced a twenty-one chromosome genome from the seven chromosome ancestor. It is not too difficult to reconstruct the general structure of the immediate post- *γ* chromosomes since they differ from the nineteen-chromosome grape genome in only four or five large rearrangements. This baseline genome is the ultimate resource for deducing gene-order change in the core eudicots, serving as a close proxy for the ancestral hexaploid [18].

The main data for this paper consists of three-way synteny blocks between grape, *Rhazya* and coffee. As a first step, we used SYNMAP,

https://www.genomevolution.org/CoGe/SynMap.pl

(with default parameters) in the CoGe platform

https://genomevolution.org/CoGe/

to find pairwise synteny blocks between grape and *Rhazya* (806 blocks), between *Rhazya* and coffee (583 blocks), and between coffee and grape (722 blocks), drawing on the CDS sequences for these genomes also stored in CoGe.

In this construction, we used overall gene similarity and *K*_*s*_ scores to establish thresholds for distinguishing duplicate gene pairs due to speciation (orthologs) which were retained for our analysis, from the older, weaker and sparser gene pairs due to the gamma triplication (“out-paralogs”), which were discarded. We coloured (i.e., labelled) the blocks according to the 21 ancestral core eudicot chromosomes [18], and noted the strand (polarity) of the block in the two genomes.

The *Rhazya* genome sequence assembly [15] is made up of 980 scaffolds, 731 of which do not contain any genes. Among those that contain genes, many do not contain enough of them to establish synteny blocks with both grape and coffee. Others contained only one synteny block entirely contained in a grape-coffee block. None of these contain information helpful to describe the evolutionary divergence of *Rhazya* at the synteny block level from the other genomes. What is required are *Rhazya* scaffolds containing at least two successive synteny blocks that are not successive in either coffee or grape or both. Ultimately only 22 *Rhazya* scaffolds were large enough to satisfy this criterion. All of these contained at least 200 genes.

To construct the three-way blocks, we then sorted all the pairwise synteny blocks according to their position on the *Rhazya* genome. Because of the relatively small size of the *Rhazya* scaffolds, the coffee-grape blocks contained virtually no breakpoints that would further split any of the *Rhazya* blocks into two blocks. Some 70 triples constructed from the pairwise coffee-*Rhazya* and grape-*Rhazya* blocks were clearly coherent, usually missing at most a few genes at the start or end of each block, because of widespread fractionation. For almost twice as many, different parts of a *Rhazya* region were syntenic with two or more regions in coffee, in grape, or sometimes in both, resulting in two or more new contiguous syntenic blocks in *Rhazya* but non-contiguous blocks in one or the other or both of the other genomes. Parts of contiguous blocks in the other genomes homologous to non-contiguous parts in *Rhazya* would be determined in an analogous way.

Finally 204 three-way blocks were identified by this procedure, and formed the initial data set for our ancestral genome reconstruction. Note that these blocks are defined by pairwise orthologies between the genomes, and may involve very few orthologous triples, or none at all. Note as well that it is possible to use two-way blocks in addition to the three-way blocks in the type of analysis we undertook, and this is fairly reliable in identifying blocks in the ancestor, but it is not very useful in establishing their genomic position.

In this process, although conflicting paralogous identifications occurred at the level of individual gene pairs, these were not reflected in any confusion at the synteny block level. Within these blocks, the number of gene pairs and the average similarity levels between descendants of the same *γ* subgenome were clearly greater than between descendants of different subgenomes, and gene duplicates generated after *γ* had no effect. Such duplicates were either detected as tandem duplicates and removed automatically by SynMap or, isolated from syntenic context, did not show up at all in syntenic analysis.

We characterized this data set as 0-level resolution, because no data were excluded. In addition, we repeated the entire reconstruction at resolution levels *L*=10,20,30,40,50,60 and 70, at each level simply ignoring all blocks with less than *L* genes. Note that higher *L* corresponds to less resolution. In the figures presented here, the axis representing resolution is labelled “ ← resolution” to indicate that lower values of *L* indicate higher resolution.

Whenever removing smaller blocks from consideration revealed pairs of remaining blocks close together on all three genomes, i.e., with less than 250 genes (including those genes no longer considered to be in blocks) separating them on all three, we combined them. Because of the conservatism of the grape genome chromosomes, these combined blocks were virtually always of the same core eudicot “colour”, but their gene orders, or polarity of the blocks, were not always consistent. We found that this “same-colour” tendency holds for a cutoff of 250 or less, but broke down if we merged blocks 300 genes or more apart. On the other hand, setting a cutoff less than 250 meant that large neighbouring blocks of the same colour sometimes failed to merge. Table 1 summarizes the the number of remaining blocks after small-block deletions and mergers, at each resolution, and the total number of genes in the remaining blocks.

**Table 1:** Loss of gene content from blocks retained as resolution decreases (as *L* increases)

Level *L*	Blocks	Blocks	After	Genes Not	Genes
	Deleted	Remaining	Mergers	included	Remaining
0	0	204	162	0	13862 (100%)
10	11	193	149	264	13599 (98%)
20	36	168	118	853	13009 (94%)
30	59	145	92	1550	12312 (89%)
40	75	129	76	2167	11695 (84%)
50	85	119	62	2689	11173 (81%)
60	91	113	56	3084	10778 (78%)
70	97	107	51	3546	10317 (74%)

To resolve the problem of conflicting orientations of merged blocks, the ensuing analysis was carried out repeatedly, each time changing the sign of one of the indeterminate blocks, keeping the change if it improved the objective function, until no further improvement was observed. Because neighbouring blocks in the original construction, where *L*=0, could also be merged under the same “colour" criterion", the same procedure was also carried out at this level.

At each level of resolution, Xu’s median solver for DCJ [19] was employed to infer the Gentianales ancestor. In producing an exhaustive list of medians, this was efficient enough to carry out several cycles of the polarity assignment described above.

For each median, we could then calculate the DCJ distance [17] to each of the three genomes, using a stepwise approach to reducing the cycle graph, prioritizing either translocations or reversals.

Table 2 shows the DCJ distance between the pairs of genomes, each represented as a series of blocks on chromosomes, at all resolution levels, as well as the same measure applied to the content of each block, and then summed. The latter is not directly pertinent to this paper, but is included for general interest. The table also displays the average positioning of the median with respect to the input genomes.

**Table 2:** Properties of rearrangement and median analyses. *m*= median, *d*= DCJ distance

Level	Distance between genomes		Average	Average	Average	Median
*L*	Block-internal	Whole blocks	*d*(*m*,*Rhazya*)	*d*(*m*,coffee)	*d*(*m*,grape)	Distance
0	68	322	74.5	38.6	58.9	172
10	72	293	68.5	34.0	54.5	157
20	88	237	52.5	34.1	42.4	129
30	92	186	38.7	31.1	34.2	104
40	92	148	31.0	21.0	28.0	80
50	100	116	27.6	14.3	22.1	64
60	100	105	21.5	16.9	19.6	58
70	96	97	18.9	1.0	19.6	40

We also carried out the same calculations in the framework of the breakpoint distance. In the case of the breakpoint median, our method used a maximum weight matching algorithm [20].

Finally, we calculated the minimum number of translocations from the median to the three plant genomes according to a very conservative probabilistic model [1, 2].

## Results

**Fig. 1:**
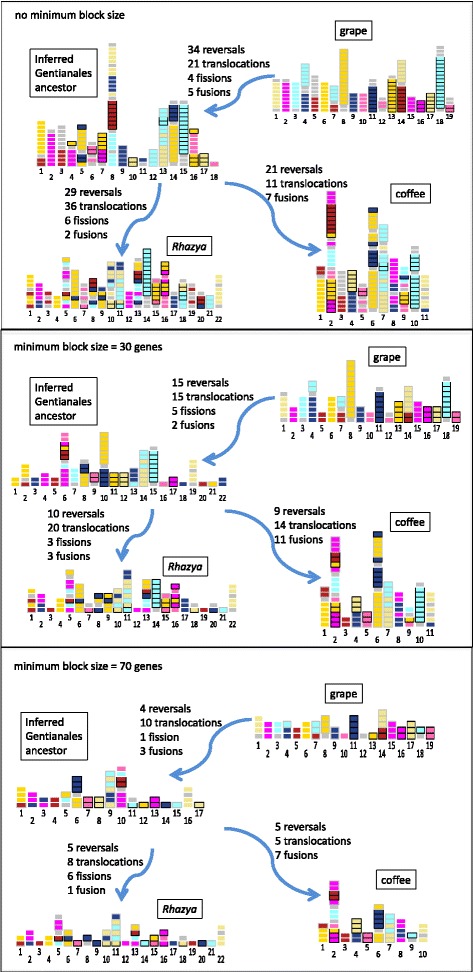
Inference of Gentianales ancestor at 3 levels of resolution. Grape genome is proxy for the ancestral core eudicot genome. Colours reflect 7 pre- *γ* chromosomes; triple homeologous copies distinguished by black, white, and no borders. Grey blocks reflect regions of uncertain homeology. Uniform size blocks in each scheme not scaled by number of genes. For individual block identification, see http://albuquerque.bioinformatics.uottawa.ca/Softwares/resolution/index.html

Figure 1 depicts the results of the median construction at three levels of resolution. The median is not unique (see “The set of medians” section below), but we used typical ones for these displays. The coloured regions reflect the seven core eudicot chromosomes before the *γ* triplication, while the three subgenomes after *γ* are distinguished by different border patterns around the same colour blocks.

Comparing the three genomic (DCJ) distances with evolutionary time (the Gentianales ancestor dating from 65 My ago, and the core eudicot represented by grape about 55 My older [21]) in the fully resolved analysis, reveals that coffee is much more conservative than *Rhazya*. As is well known, grape is extremely conservative at the level of syntenic blocks, and this is reflected by the DCJ distance from the median to grape, where the time span is about 165 My, including the time from the core eudicot ancestor to the Gentianales ancestor plus the time to the modern grape genome.

### The set of medians

Under the kind of distances used in comparative genomics, the median is generally not unique. For our eight levels of resolution, starting at level 0 and ending with level 70, the number of distinct medians was 543, 4296, 1710, 21016, 256, 324, 231 and 425.

**Fig. 2:**
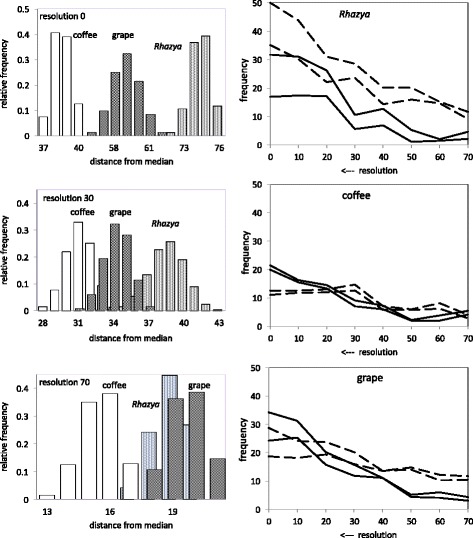
Left: Effect of resolution on distance from median to *Rhazya*, coffee and grape. From top to bottom, with decreasing resolution, histograms for the three species overlap increasingly, blurring the gene order conservatism of coffee and grape versus the extensive rearrangement propensity of *Rhazya*. Right: Effect of resolution on numbers of translocations and inversions necessary to transform median to *Rhazya*, coffee and grape. Dotted line: translocations. Unbroken line: reversals. Upper and lower lines in each colour represent the maximum and minimum number found when prioritizing or delaying the corresponding operation versus the other operation. Fusions and fissions (not shown) are found at low, comparable, levels except for coffee which shows 5-10 fusions only, with no trend according to resolution

Figure 2 shows that the evolutionary distance of each genome may vary slightly from one median to another. This variation is, however, not very large as can be seen from the low-variance distributions, and does not get worse with decreasing resolution. More important is that the distinction between the three genomes, very clear at level 0, is blurred and even reversed (for *Rhazya* and grape) at level 70.

What also changes more drastically are the proportions of translocations and inversions as the median scores decrease, also displayed in Fig. 2. At full resolution, *Rhazya* shows a large number of translocations, while coffee and, to a lesser extent, grape, show more reversals. As resolution decreases, these patterns are disrupted and even reversed. In other words, the structure of the DCJ distance in terms of translocations and reversals, is lost with decreasing resolution.

### Distances

**Fig. 3:**
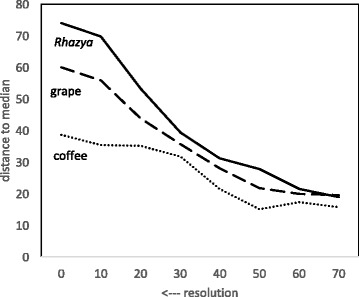
Effect of resolution on DCJ distance from median to *Rhazya*, coffee and grape

**Fig. 4:**
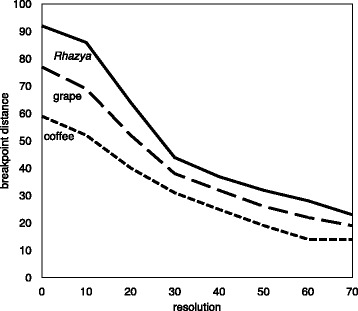
Effect of resolution on breakpoint distance from median to *Rhazya*, coffee and grape

Figures 3 and 4 depict the decrease in the total of the DCJ and breakpoint distances between the three genomes and the median calculated from these genomes. In addition, Fig. 3 shows that the relative conservatism of coffee and grape compared to *Rhazya*, very clear at full resolution, is almost completely blurred by the time level 70 is reached.

In contrast, the breakpoint distance decreases more or less proportionately for the three genomes. This simply reflects the fact that the same blocks are deleted from the three genomes at each step,

### Breakpoint reuse

In the context of genome rearrangement theory, a measure of the randomness of one genome relative to another is the statistic *r*=2*d*/*b*, where *d* is the rearrangement distance and *b* is the number of breakpoints [22–24]. As resolution of the comparison of two genomes decreases, *r* increases from a minimum value of 1 to a maximum of 2. The quantity *r* is called “breakpoint reuse” because its increase is sometimes attributed (mistakenly in most cases) to a rearrangement history where almost all reversals and translocations, instead of creating two new breakpoints in the genome, introduce only one new breakpoint in a genome, while also “reusing" an existing breakpoint created by a previous translocation. A number of studies have shown that this interpretation is illusory (e.g. [25]), an artifact of decreasing resolution creating gene orders or block orders effectively random with respect to each other in the two genomes being compared.

**Fig. 5:**
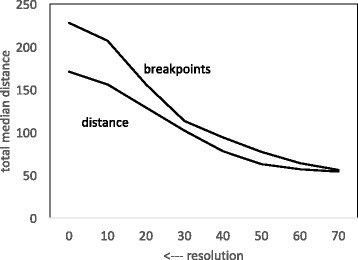
Effect of resolution on total median scores

**Fig. 6:**
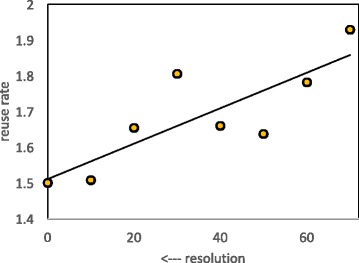
Effect of resolution on breakpoint reuse. Least-squares fit line *R*^2^=0.68

Figure 5 suggests that reuse is not constant as resolution decreases, and Fig. 6 confirms the descent (or ascent) into randomness as resolution decreases.

### Normalization

**Fig. 7:**
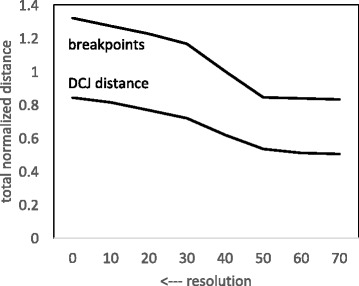
Effect of resolution on total normalized median scores

It might be thought that the decrease in distances seen in Fig. 5 could be attributable simply to the number of remaining synteny blocks as resolution decreases. The use of normalized distance has been suggested, and used successfully, in different contexts [26, 27], but in Fig. 7 we see that normalizing distances by the number of blocks involved explains only part of the decrease. The remaining decrease must be attributable to the increase of randomness of each genome with respect to the others as resolution decreases.

### A non-constructive method

If a chromosome *i* in genome B consists of many alternating blocks corresponding to just two chromosomes *j* and *k* of genome A, it is likely that this *i* arose from a translocation or a fusion of *j* and *k*, followed by several reversals or transpositions only affecting the new chromosome. If on the other hand *i* contains many blocks, each corresponding to a different chromosome in genome A, then it is more likely that *i* results from many translocations or fusions of the corresponding chromosomes of A, followed by just a few inversions, or none.

Based on the idea, we developed an inference procedure for the number of rearrangements of various kinds separating genome B from genome A [1, 2]. See also [28, 29] for further work on this theory.

At the outset, assume the first translocation on the lineage from genome A to genome B involves chromosome *i*. The mild assumption of a uniform density of breakpoints across the genome implies that for any *j* the “partner” of *i* in the translocation will be chromosome *j* with probability $p_{i}(j) = \frac {p(j)}{1-p(i)}$, where *p*(*i*) is the proportion of the genome covered by chromosome *i*.

If the translocation was with chromosome *k*, re-assign the labels *i* and *k* arbitrarily to the two new chromosomes. Thus the probability that the new chromosome labelled *i* contains no fragment of genome A chromosome *j*, where *j*≠*i*, is 1−*p*_*i*_(*j*). For small *t*^(*i*)^, after chromosome *i* has undergone *t*^(*i*)^ translocations, the probability that it contains no fragment of the genome A chromosome *j* is approximately $(1-p_{i}(j))^{t^{(i)}}$, neglecting second-order events, for example, the event that *j* previously translocated with one or more of the *t*^(*i*)^ chromosomes that then translocated with *i*, and that a secondary transfer to *i* of material originally from *j* thereby occurred.

Then the probability that the genome B chromosome *i* contains at least one fragment from *j* is approximately $1 - (1 - p_{i}(j))^{t^{(i)}}$. Let *c*^(*i*)^ be the number of genome A chromosomes with at least one fragment on *i*, i.e., the number of conserved syntenies on chromosome *i*, so that

1 $$ c^{(i)}\le c,  $$

where *c* is the total number of chromosomes. Then

2 $$ E\left(c^{(i)}\right) \approx 1 +\sum_{j\ne i} \left[1 - (1 - p_{i}(j))^{t^{(i)}}\right]  $$

so that

3 $$ c - E\left(c^{(i)}\right) \approx \sum_{j\ne i} (1 - p_{i}(j))^{t^{(i)}}  $$

where the leading 1 in (2) counts the fragment containing the left-hand endpoint of the genome A chromosome *i* itself. Suppose there have been a total of *t* translocations in the evolutionary history. Then, $\sum _{i} t^{(i)} = 2t$ and we can expect these to have been distributed among the chromosomes approximately as *t*^(*i*)^=2*tp*(*i*) so that

4 $$ c^{2} - \sum_{i} E\left(c^{(i)}\right) \approx \sum_{i}\sum_{j\ne i}(1 - p_{i}(j))^{2tp(i)}.  $$

Substituting the *c*^(*i*)^ for the *E*(*c*^(*i*)^) in Eq. (4) suggests solving

5 $$ c^{2} - \sum_{i} c^{(i)} = \sum_{i}\sum_{j\ne i}(1 - p_{i}(j))^{2\hat{t}p(i)}  $$

to provide an estimator of *t*.

For the case where A and B have different numbers of chromosomes *c* and *d*, and where *q*(*j*) is the proportion of B taken up by chromosome *j*, a better model is

6 $$ cd-\sum_{i} c^{(i)}=\frac{d-1}{d}\sum_{i} \sum_{j} (1-q(j))^{2p(i)\hat{t}}.  $$

If, as with the plant genomes, chromosome lengths are not too variable within a genome, this reduces to

7 $$ 1-\frac{\sum_{i} c^{(i)}}{cd}=\left(1-\frac{1}{d}\right)^{1+2\hat{t}/c}.  $$

**Fig. 8:**
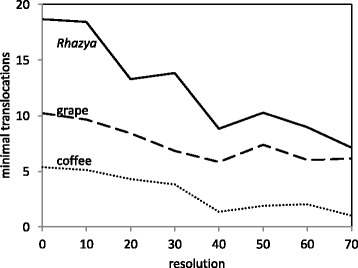
Effect of resolution on (minimal) translocation number from median to *Rhazya*, coffee and grape

The results of solving this are shown in Fig. 8. Given the high reuse rate for these comparisons, we omitted the factor 2 in solving Eq. (7). This only affects the scale of the y-axis in Fig. 8.

Although the absolute numbers are clearly underestimates, in comparison with Fig. 2, the relative tendencies are the same.

## Discussion

This study only shows one side of the coin. The obverse would trace the effect of *increasing* the resolution, testing the consequences of increasing noise in the data. This could be done by changing the default criterion in CoGe for the number of genes required to establish a synteny block from 5 to 4 or even 3. Experience shows that this would allow an increasing proportion of spurious synteny blocks into the data, especially in plant genomes where co-linear paralogs from whole genome duplication and triplication abound.

## Conclusions

We have shown the multifaceted consequences of the loss of resolution in a typical comparative genomic study of three plant genomes. We reconstructed, at the synteny block level, the ancestral genome of the order Gentianales, based on coffee, *Rhazya stricta*, and outgroup grape. We showed how decreased resolution blurs the differences between the genomes, with respect to evolutionary rate and mutational process. We also showed how reuse rate increases, confirming the randomization of the genomes with respect to each other as resolution decreases.

## Abbreviations

DCJ: Double cut and join
*K*_*s*_: the number of synonymous substitutions per synonymous site
